# Who Protects Youth from Suicide After Bullying? Comparative Effects of Family, Peer and Teacher Support in Spanish Adolescents

**DOI:** 10.1007/s40653-025-00801-2

**Published:** 2025-12-18

**Authors:** Héctor Galindo-Domínguez, Daniel Losada Iglesias

**Affiliations:** 1 Departamento de didáctica y organización escolar, Universidad del País Vasco/Euskal Herriko Unibertsitatea, UPV/EHU, Juan Ibáñez de Santo Domingo Kalea, 1, Vitoria-Gasteiz, 01006 Spain; 2 Departamento de didáctica y organización escolar, Universidad del País Vasco/Euskal Herriko Unibertsitatea, UPV/EHU, Plaza Oñati, 3, Donostia-San Sebastian, 20018 Spain

**Keywords:** Social support, Adolescents, Suicidal ideation, Bullying, Victimization

## Abstract

This study analyzed the moderating role of different sources of social support in the association between bullying victimization and suicidal ideation among adolescents. Participants were 898 Spanish adolescents, who completed validated measures of school victimization, perceived family, peer, and teacher support, school environment, and suicidal risk. The findings indicated that the link between bullying victimization and suicidal ideation was stronger in females than in males. Family support generally showed a protective function for females; however, under high victimization, it exerted a comparatively stronger protective effect for males. Peer support was more effective in reducing suicidal ideation among females and among students who had not repeated a grade. Teacher support emerged as a consistent protective factor across sex, age, and grade-retention status, contributing to lower suicidal ideation regardless of individual characteristics. Overall, the results underscore the importance of considering the differentiated contributions of family, peers, and teachers when addressing the psychological impact of bullying. They also highlight the need to reinforce supportive interpersonal contexts in educational settings to mitigate suicidal ideation, one of the leading causes of mortality in adolescence.

## Highlights

- The role of social support in preventing suicidal thoughts among bullying victims is still poorly understood.
- Bullying victimization has a stronger effect on suicidal thoughts in females compared to males.
- Overall, Family Support helps females more in lowering suicidal thoughts, but in bullying situations, higher Family Support benefits males more than females.
- Peer Support is more effective in reducing suicidal thoughts among females and students who have not repeated a course.

## Introduction

### Suicidaion as a Consequence of Bullying Victimizationl Ideat

Suicide is the third highest cause of mortality in adolescents, happening 90% of the times among teenagers of low- or middle-income countries (World Health Organization, 2020). Suicidal ideation and behavior have turned into a public burden, necessitating significant effort to dive into our understanding of how, why, and when it might be averted in light of this circumstance (Hinduja & Patchin, 2018).

Due to the negative occurrences that are having place in the last years, the literature is beginning to focus on the association between bullying and suicide (Hertz et al., 2013). Bullying is considered a subcategory of peer violence with specific characteristics, like intentional behavior, repetition and power imbalance (Rodrigues et al., 2020), and normally has four different types of involvement: victims, perpetrators, victim-perpetrators and witnesses (Monteiro et al., 2017).

It is noteworthy the large amount of meta-analysis available in the literature that attempt to shed some light on the prevalence of suicidal thoughts and behaviors in adolescents bullying victims. Firstly, a meta-analysis carried by Kim and Leventhal (2008) reveal that the prevalence of suicidal ideations is higher (OR 1.4–10.0), in bullying victims regardless of belonging to general students or belonging to students with some minority characteristics, such as Asperger syndrome cases or sexual minority cases, to cite a few. These results have been fairly unanimously shared by those shown in other cross-sectional (OR 1.4–10.0) and longitudinal studies (OR 1.7–11.8) that show the significant association and prevalence of suicidal ideations and suicidal attempts in adolescent bully-victims (Brunstein et al., 2010; van Geel et al., 2014).

Recently, Holt (2015) confirmed that being involved in a bullying case is associated with suicidal ideations and behaviors (OR 2.12 to 4.02), and compared to adolescents not involved in bullying cases, they present higher values of suicidal ideations (Yen et al., 2015). This finding was also shared years later by Baiden et al. (2019), who state that the experience of bullying victimization was a significant predictor of both suicidal ideation and suicide attempt. In fact, past longitudinal research on the subject reveal that there may be a considerable causal association between victimization and low well-being, as manifested by severe depression or suicidal thoughts (e.g., Brunstein et al., 2010; Ttofi et al., 2011).

By the same token, the relationship between bullying victimization and suicidal ideation is affected by a large number of personal, cognitive and social characteristics. For instance, in some cases, the prevalence of suicidal ideation in bullying victimization cases is promoted by a poor teachers and family support (e.g., Miranda et al., 2019), by a poor well-being (e.g., Baiden et al., 2019; Cosma et al., 2017; Moore et al., 2017; Weng et al., 2017), by a low emotional intelligence (Galindo-Domínguez & Losada, 2023), by the presence of interpersonal difficulties or lack of friends (e.g., Acquah et al., 2016; Baiden et al., 2019; Galindo-Domínguez & Losada, 2023; Saeed & Taremian, 2020) or by a poor life satisfaction (Galindo-Domínguez & Losada, 2023; Miranda et al., 2019; Weng et al., 2017). Furthermore, the literature has examined the impact of individual characteristics such as sex (Brunstein et al., 2010; Lucas-Molina et al., 2018; Reed et al., 2015; Secord & Demaray, 2018) or age (Alavi et al., 2017; Reed et al., 2015) on the relationship between bullying victimization and suicidal ideation in cross-sectional and longitudinal studies.

Finally, it should be mentioned how the consequences of bullying cases are so significant that Reid et al. (2016) demonstrated how having been bullied in childhood can have consequences years later. More specifically, it was appreciated how having suffered bullying victimization in childhood was significantly associated with depression and anxiety years later.

### Relevance of Social Support for Preventing Suicidal Ideation

Social support can be understood as the feeling that an individual has to think that others love and care for him or her, as well as the feeling that it causes to one when it is appreciated and integrated into a network of human connections (Agbaria & Bdier, 2020).

The relevance of this variable has been analyzed in longitudinal studies, observing how different types of external agents to the subject can help reduce suicidal ideation in bullying victimization cases (e.g., Nguyen et al., 2019). When these agents are not presented negative effects could happen. In this sense, isolation can reduce access to social support from families and friends, thus reducing levels of perceived social support, causing loneliness and becoming a risk factor that can cause depressive symptoms, anxiety or even an increase in suicidal ideations (Zhou et al., 2020).

Indeed, this is the main idea reflected behind the buffering hypothesis of social support that suggests that when social support is available, it is assumed that it contributes to reducing and weakening the negative impact of stressors (Cohen & McKay, 1984). The buffering effect of social support can materialize in two different ways. First, bearing in mind that social support will be available in times of crisis allows for the improvement of coping skills. Second, in cases where a major stressor is present, social support can reduce the painful consequences of that stressor, having a positive impact on the subject’s health or behavior. (Cohen et al., 2000).

As it has been observed, social support could serve as a protective factor in the relationship between victimization and negative outcomes, like stress, anxiety, or suicidal ideation. Therefore, on the next heading the main previous findings about the moderating of social support between victimization and suicidal ideation is exposed.

### Moderating Role of Social Support in Bullying Cases

There are some studies that have analyzed the buffer effect of social support and coping between bullying victimization and stress levels, finding a significant moderation effect of social support and coping strategies between these variables (e.g., Konishi & Hymel, 2009). This finding was also highlighted in more recent studies pointing out a moderation effect of perceived social support in the relationship between victimization and suicidal ideation (Bonano & Hymel, 2010; Liu et al., 2017). This conclusion, as stated by Bonano & Hymal (2010, p. 420), bears the idea that “the more socially hopeless someone becomes, the greater is their risk for having suicidal thoughts’’. Moreover, Eze et al. (2021) also found a significant buffer effect of social support between victimization and suicidal ideation. Specifically, when compared to adolescents with moderate or high social support, the positive association between victimization and suicidal thoughts was largest for those with poor social support. This finding emphasizes the importance of social support and social climate as a key variable when reducing negative outcomes (Dorio et al., 2020; Ringdal, 2020; Scardera et al., 2020). In addition, this finding has been replicated in other fields and with another type of samples. For instance, Birkeland et al. (2020) in their study, point out how social support plays a significant moderation effect between workplace bullying and mental distress for both males and females.

Other studies have focused on analyzing the buffering effect of social support on academic performance in bullying cases. For instance, Rothon et al. (2011) revealed how bullied adolescents were less likely to achieve the appropriate academic achievement benchmark for their age group. One possible explanation for this finding is that those students who have suffered bullying are more likely to play truant or be absent from school as a way of avoiding the negative consequences of school (Smith et al., 2004). In order to avoid this situation, recent studies have demonstrated that students, more likely in girls than in boys, who receive higher peer support are more prone to report a victimization case (Coyle et al., 2021).

As a manner of avoiding this victimization, especially family and friends play a fundamental role as it was appreciated that a significant level of social support was important to improve the adolescent’s mental health. What is more, Rothon et al. (2011) demonstrated how high levels of social support from friends and moderate (not high) levels of social support from family were able to protect bullied adolescents from underperforming. As stated by Rothon et al. (2011), while some suggest that parental support is necessary for teenage resilience development, overprotective parenting may have the opposite impact, making young people less assertive and independent. A similar finding has been again obtained in recent studies with adolescents pointing out how a high family support in situations of school performance stress can cause the contrary effect that was expected, worsening students’ mental wellbeing (Ringdal, 2020). This finding explains why teenagers spend an increasing amount of unsupervised time with their friends rather than their families, gradually gaining their independence from their parents. Hence, social support from peers and friends could be more relevant for adolescents than social support from family (Rothon et al., 2011). However, there is no definitive evidence as other studies suggest that all forms of social support are related to lower violent behavior (Šmigelskas et al., 2018). Finally, noteworthy are Secord’s et al.’s (2018) findings which highlight that parent, classmate, and close peer support buffered the association between depression and suicidal ideation.

### Purpose of the Study

Based on the more recent literature, several clear research gaps have been identified. First, Holt (2015) emphasizes the importance of how future studies should highlight the implications that mental health can have in bullying cases for the prevention of suicidal ideations and behaviors. Despite this, empirical research explicitly linking mental health indicators with the protective role of social support in bullying contexts remains limited. This idea has been recently echoed in Quintana-Orts et al. (2021), claiming that although the data supported the hypothesized relationship between traditional victimization, isolation, and suicidal ideation, this association should be investigated on a broader scale, incorporating additional social and emotional factors (e.g., social connectedness, social support) and examining the relative influence of self- and other-focused emotional skills to better understand interpersonal abilities as buffers against the impact of traditional bullying.

Second, some studies have highlighted the scarcity of research analyzing the relationship between peer, family, and school support and adolescent suicide (Miller et al., 2015), as well as peer and family support in the context of bullying victimization (Shaheen et al., 2019). This indicates a need to integrate multiple sources of social support simultaneously rather than focusing on isolated domains. Third, authors have pointed out the necessity of performing more complex statistical analyses, such as moderation analyses, that could explain the differential impact of various domains of social support (Lee et al., 2022, p. 7).

Finally, there is a scarcity of studies that consider the different types of social support in a disaggregated way, particularly when combined with individual characteristics such as age or sex. Nonetheless, this limitation has been recently addressed by Lee et al. (2022), who point to the relevance of analyzing types of social support while considering personal variables to achieve a more nuanced understanding and to design more targeted interventions. Likewise, Palomares-Ruiz et al. (2019) emphasize the importance of separate analyses by type of social support, accounting for both internal and external adolescent variables. Overall, these gaps indicate that the moderating role of family, peer, and teacher support on the link between bullying and suicidal ideation remains underexplored, particularly in interaction with demographic and academic variables.

Based on these limitations, three key Research Questions arise:

#### RQ1

Does peer support moderate the relationship between bullying victimization and suicidal ideation in adolescents, and if so, to what extent do individual characteristics (age, sex, or retaking a course) influence this moderation?

#### RQ2

Does family support moderate the relationship between bullying victimization and suicidal ideation in adolescents, and if so, to what extent do individual characteristics influence this moderation?

#### RQ3

Does teacher support moderate the relationship between bullying victimization and suicidal ideation in adolescents, and if so, to what extent do individual characteristics influence this moderation?

Taking into consideration these three key research questions, the main aim of this study is to analyze whether peer, family, and teacher support moderate the relationship between bullying victimization and suicidal ideation in adolescents, accounting for the potential moderating effects of age, sex, and academic repetition.

## Methodology

### Sample

A total of 898 Spanish teenagers from the Basque Country (northern Spain) participated in the current study (Age = 13.55, SD = 1.26). 188 (20.9%) attended public schools, while 710 (79.1%) attended subsidized schools. There were 448 males (49.9%) and 450 girls (50.1%) in the overall sample. 195 (21.7%) were in their first year of secondary school, 275 (30.6%) in their second year, 216 (24.1%) in their third year, and 212 (23.6%) in their fourth year. 134 (14.9%) of the entire sample had retaken a year at least once.

### Instruments

Data was gathered using four distinct self-assessment measures. The first one was related to a number of personal variables, such as sex, age, course, if he or she has retaken a year… As previously collected, the current literature has already highlighted the relevance of sex or age on the relationship between bullying victimization and suicidal ideation, but we wanted to introduce the variable of retaking a year due to the fact that this kind of students could perceive lower social support values, especially from teachers and peers, as they are out of their comfort zone in a new class without their lifelong friends.

The *Peer School Victimization Scale* was the second questionnaire used to assess peer victimization (Cava & Buelga, 2017). This scale is made up of 12 items that assess how frequently specific peer victimization situations occur. Indeed, according to the definition of bullying offered, recurrence is one of its primary characteristics; so, in certain situations, this victimization might be deemed bullying. Furthermore, the dimensions of this scale are: Verbal violence (e.g., A colleague has made fun of me), Physic violence (e.g., A colleague has pushed me hard) and Relational violence (e.g., A colleague has ignored me or left me aside to make me feel bad). It is graded on a 5-point Likert scale, with 1 being the worst (it has never happened to me) and 5 being the best (it happens to me very often). Furthermore, the authors’ given psychometric characteristics demonstrated a good model fit (CFI = 0.991; TLI = 0.988; RMSEA = 0.052; X^2^ = 112.94; df = 41; p.001).

The third instrument used focused on measuring the perceived social support was the *Scale of Perceived Social Support* (Arechabala & Miranda, 2002). This scale is formed by 12 items and the following dimensions: Family Social Support (e.g., I am sure that my family tries to help me.) and Peer Social Support (e.g., I can count on my friends when I have problems). It is measured using a 5-point Likert scale ranging from 1 (totally disagree) to 5 (totally agree). The psychometric properties provided by the authors of the scale revealed a good model fit (CFI = 0.904; GFI = 0.85; X^2^ = 7.6; *p* <.01). In addition, in order to measure the perceived social support from teachers *Interpersonal Social Climate* dimension was used from the Spanish Adaptation of School Environment Scale (Villa, 1992). This dimension is formed by 4 items that measure the perception that students have about the degree of closeness and understanding shown by the teacher for the emotions and concerns of the students (e.g. My teacher spends a lot of time helping us with our school work and with our personal problems). Moreover, this dimension is measured using a 5-point Likert scale ranging from 1 (totally disagree) to 5 (totally agree).

The final instrument used to measure suicidal thoughts was the Suicide Risk Inventory for Adolescents - A Version (Hernández & Lucio, 2006). This instrument consists of 25 items and the following dimensions: Suicidal Ideation (e.g., I have thought about committing suicide), life satisfaction (e.g., In my life there are good moments), interpersonal difficulties (e.g., I prefer to be alone) and difficulties in school (e.g., my teachers ignore me). It is assessed on a 5-point Likert scale, ranging from 1 (totally disagree) to 5 (totally agree). The psychometric parameters of the scale were reported years later by Alarcón-Vásquez et al. (2019), indicating an outstanding model fit (CFI = 0.980; X^2^ = 1105.04; df = 249; RMSEA = 0.080; TLI = 0.970; AIC = 1278.18).

The time frame considered for all the scales (peer victimization, social support and suicidal ideation) was 1 month, due to the fact that it is thought to be an adequate period of time in which students are able to remember the most significant events that have happened to them (more distant periods of time could be more complex to remember, and therefore to evaluate), as well as being a sufficient period of time in which both positive and negative phenomena can occur (shorter periods of time could be worse due to the lack of frequency of the phenomenon being studied). Previously, this time frame has been used by other studies (Rodrigues et al., 2020).

### Procedure

The procedure began with the creation of a database of possible participating schools. The educational centers in the Basque Country (Spain) were chosen because they were close to the researchers. After completing the database, the authors contacted the management teams through email, and those who were interested were supplied the necessary documents so that they could decide whether or not to join (family informed consent and questions students were going to answer). The centers were given a two-month response period to arrange informed consents with families and answer to the questionnaire. During this time, students at school completed the various scales online using Google Forms. All questions were collected anonymously and privately, in accordance with the ethical norms of these studies.

### Data Analysis

The data analysis was carried out step by step in a process of three phases. Firstly, descriptive statistics were analyzed. On this matter, means and standard deviations from the main dimensions were studied. This analysis was complemented by analyzing correlations between the main dimensions using Pearson’s r and studying internal consistency values using Cronbach’s alpha. Straightaway, using *Process* for *SPSS Statistics 25*, three moderated moderation analyses were carried out. Specifically, interactions between the main variables, as well as conditional effects were studied. For these analyses, victimization was considered as explanatory variable, suicidal ideation as outcome variable, and different types of social support, as well as individual characteristics as moderation variables. The points of the moderating variable were calculated using the *pick-a-point* technique (16th, 50th and 84th percentiles).

## Results

### Results of the Descriptive Analyses

First, as shown in Table 1, a descriptive analysis was performed, which included the examination of means, standard deviations, correlations, and Cronbach’s alpha values. According to the means analysis, the most common kind of victimization was verbal victimization (M = 2.09; SD = 0.990), followed by physical victimization (M = 1.56; SD = 0.729) and relational victimization (M = 1.68; SD = 0.874). With regard to social support, adolescents perceived a higher social support from their families (*M* = 4.07; *S*D = 0.880) and friends/peers (*M* = 3.99; *SD* = 0.997) compared to the social support perceived by their teachers (*M* = 3.38; *SD* = 1.11). Finally, life satisfaction was fairly high (M = 3.93; SD = 0.791), but school problems (M = 2.16; SD = 0.869) and interpersonal issues (M = 2.40; SD = 0.896) were moderated. Suicidal thoughts were fairly low (M = 1.94; SD = 0.998).

**Table 1:** Descriptive analysis

	M	SD	1	2	3	4	5	6	7	8	9	10
VBV	2.09	0.990	(0.877)	0.616**	0.637**	− 0.096**	− 0.169**	− 0.200	0.351**	− 0.184**	0.374**	0.305**
PHV	1.56	0.729		(0.859)	0.350**	− 0.016	− 0.093**	− 0.150**	− 0.232**	− 0.078*	0.240**	0.264**
REV	1.68	0.874			(0.834)	− 0.109**	− 0.166**	− 0.269**	0.378**	− 0.272**	0.393**	0.215**
TES	3.38	1.11				(0.920)	0.369**	0.171**	− 0.100**	0.278**	− 0.130**	− 0.254**
FAS	4.07	0.880					(0.890)	0.618**	− 0.427**	0.719**	− 0.452**	− 0.272**
PES	3.99	0.997						(0.906)	− 0.283**	0.475**	− 0.341**	− 0.144**
SUI	1.94	0.998							(0.899)	− 0.555**	0.740**	0.337
LST	3.93	0.791								(0.870)	− 0.566**	− 0.271**
IPD	2.40	0.896									(0.783)	0.450**
DIS	2.16	0.869										(0.538)

Note. *VBV* Verbal Victimization, *PHV* Physical Victimization, *REV* Relational Victimization, *TES* Teacher Support, *FAS* Family Support, *PES* Peer Support, *SUI* Suicidal Ideation, *LST* Life Satisfaction, *IPD* Interpersonal difficulties, *DIS* Difficulties in School. Cronbach’s alpha values are shown in the main diagonal. ** *p* <.01; * *p* <.05

With regard to correlations, the great majority of them were statistically significant, and many of them were above *r* =.50, suggesting the existence of a moderate or fairly strong association. This was the case of the correlation between verbal victimization and physical victimization (*r* =.616; *p* =.000), verbal victimization and relational victimization (*r* =.637; *p* =.000), family support and peer support (*r* =.618; *p* =.000), family support and life satisfaction (*r* =.719; *r* =.000), life satisfaction and suicidal ideation (*r* = −.555; *p* =.000), interpersonal difficulties and suicidal ideation (*r* =.740; *p* =.000) and lastly, interpersonal difficulties and life satisfaction (*r* = −.566; *p* =.000).

Finally, the Cronbach’s alpha values were examined. Except for the ‘Difficulties in School’ dimension (α = 0.538), all dimensions on this issue had a good internal consistency (α > 0.70). Because this value was lower than the stated limit, and because it was a factor with just three items, it was decided to exclude it from the study. Previous research has likewise reached this conclusion (Alarcón-Vásquez et al., 2019).

### Results from the Moderation Analysis

**Fig. 1:**
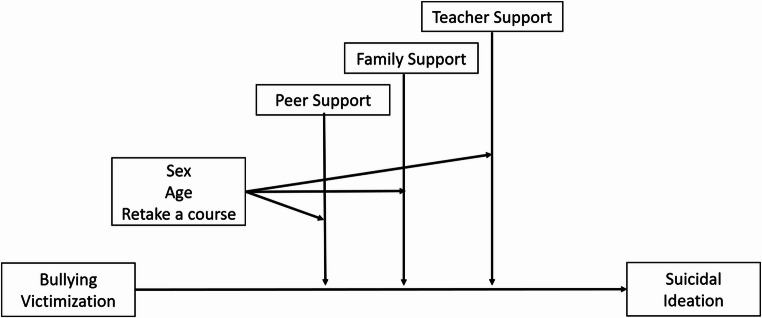
Visual diagram of the proposed moderated moderation analysis

In order to response to the different research questions, three different moderated moderation analyses were carried out. Bullying victimization was considered an explanatory variable, different forms of social support were considered moderator factors, and individual characteristics (sex, age, and course retake) were considered moderated moderator variables. Finally, suicidal ideation was taken into account as an outcome variable. Fig. 1 depicted the diagram of this analysis.

First, with regard to the RQ1, it said:

Does peer support moderate the relationship between bullying victimization and suicidal ideation in adolescents, and if so, to what extent individual characteristics, like age, sex or retaken a course, affect this relationship?

**Fig. 2:**
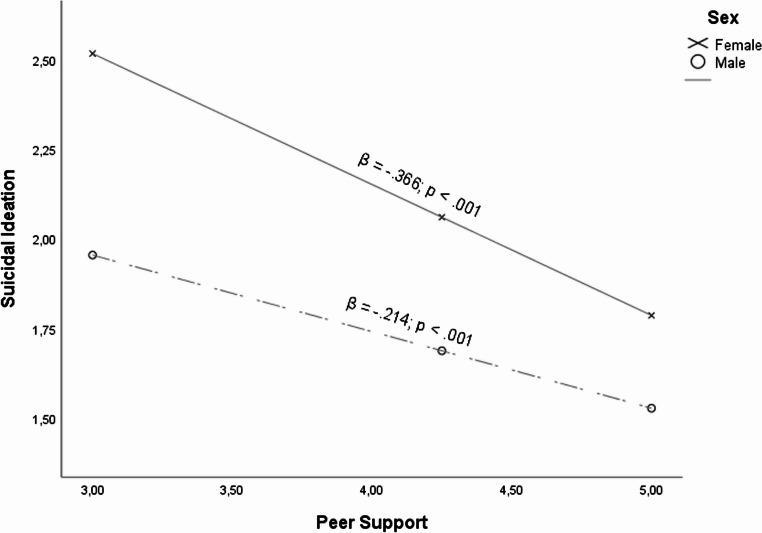
Relationship between Peer support and suicidal ideation when sex as moderator

Visual Diagram of the Proposed Moderated Moderation Analysis On this matter, from the moderation analysis collected in Table 2, it can be seen that the relationship between peer support and suicidal ideation was moderated by the sex of the adolescents (β = 0.320; *p* =.029). As illustrated in Fig. 2, despite the fact that for both sexes peer support was relevant in order to reduce suicidal ideation, it was observed from the conditional effects that for females (β = − 0.366; *p* <.001) peer support is more relevant than for males (β = − 0.214; *p* <.001) when it comes to reducing suicidal ideation.

**Table 2:** Results from the moderation analysis

Path	β	*p*	SE	LLCI	ULCI	θ
Common Interactions						
Vic * Sex	− 0.182	0.028	0.083	− 0.345	− 0.019	Female (β = 0.609; *p* <.001) Male (β = 0.427; *p* <.001)
Vic * Age	− 0.027	0.427	0.034	− 0.093	0.039	
Vic * RTK	− 0.095	0.332	0.098	− 0.289	0.098	
Interactions when Peer Support as moderator						
PES	− 0.283	< 0.001	0.032	− 0.346	− 0.220	
PES*Sex	0.320	0.029	0.147	0.032	0.607	Female (β = − 0.366; *p* <.001) Male (β = − 0.214; *p* <.001)
Vic*PSS*Sex	− 0.112	0.104	0.069	− 0.246	0.023	
PES*Age	− 0.015	0.789	0.056	− 0.126	0.096	
Vic*PES*Age	0.015	0.597	0.028	− 0.040	0.070	
PES*RTK	0.327	0.047	0.168	− 0.003	0.658	No (β = − 0.331; *p* <.001) Yes (β = − 0.014; *p* =.843)
Vic*PES*RTK	− 0.038	0.614	0.076	− 0.187	0.110	
Interactions when Family Support as moderator						
FAS	− 0.485	< 0.001	0.034	− 0.552	− 0.418	
FAS*Sex	0.571	< 0.001	0.158	0.261	0.881	Female (β = − 0.582; *p* <.001) Male (β = − 0.320; *p* <.001)
Vic*FAS*Sex	− 0.209	0.011	0.081	− 0.368	− 0.049	Female (β = 0.041; *p* =.402) Male (β = − 0.168; *p* =.010)
FAS*Age	0.077	0.222	0.063	− 0.046	0.200	
Vic*FAS*Age	− 0.040	0.213	0.032	− 0.103	0.023	
FAS*RTK	− 0.074	0.673	0.175	− 0.418	0.270	
Vic*FAS*RTK	0.143	0.088	0.084	− 0.022	0.308	
Interactions when Teacher Support as moderator						
TES	− 0.090	0.003	0.030	− 0.148	− 0.031	
TES*Sex	0.217	0.114	0.137	− 0.052	0.485	
Vic*TES*Sex	− 0.078	0.253	0.068	− 0.212	0.056	
TES*Age	− 0.038	0.488	0.055	− 0.146	0.070	
Vic*TES*Age	0.019	0.495	0.028	− 0.036	0.074	
TES*RTK	− 0.073	0.667	0.169	− 0.405	0.259	
Vic*TES*RTK	0.085	0.280	0.079	− 0.070	0.240	

*NOTE.* Vic, bullying victimization; TES, Teacher Support; RTK, retake a course; Conditioning values selected by *pick-a-point technique* (16th, 50th and 84th percentiles)

**Fig. 3:**
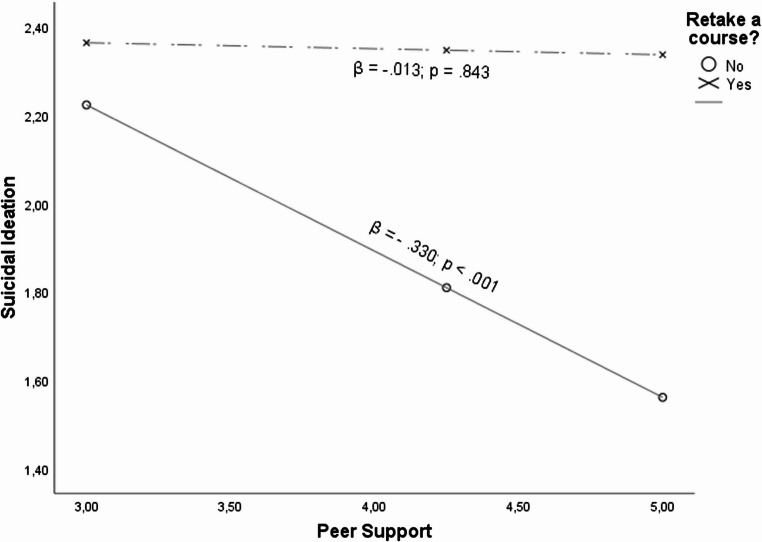
Relationship between Peer support and suicidal ideation when retake a course as moderator

Likewise. as observed in Table 2, the relationship between peer support and suicidal ideation was moderated by the condition of retaking a course, (β = 0.327; *p* =.047). As illustrated in Fig. 3, it was observed from the conditional effects that for students that had never retaken a course, (β =−0.331; *p* <.001) having a high level of peer support is crucial for reducing their suicidal ideation in contrast to students that had retaken a course, (β = − 0.015; *p* =.843).

Second, with regard to the RQ2, it said:

Does family support moderate the relationship between bullying victimization and suicidal ideation in adolescents, and if so, to what extent individual characteristics, like age, sex or retaken a course, affect this relationship?

**Fig. 4:**
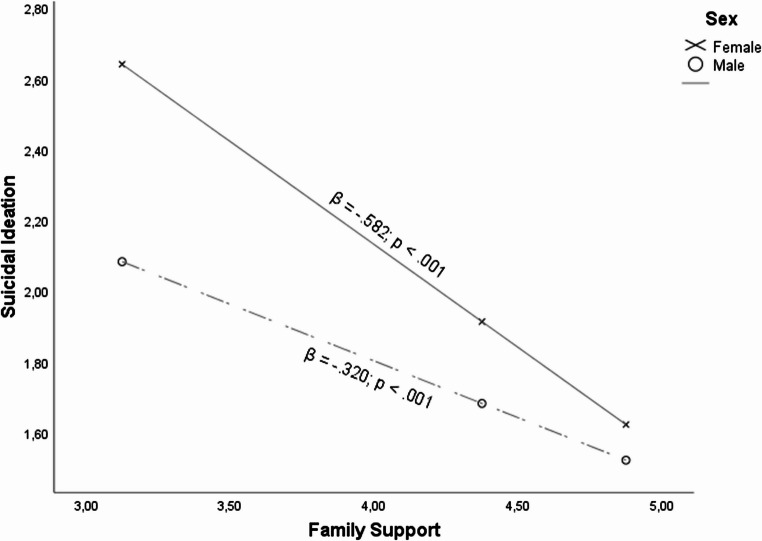
Relationship between family support and suicidal ideation when sex as moderator

On this matter, as observed in Fig. 4, it can be seen that the relationship between family support and suicidal ideation was moderated by the sex of the adolescents (β = 0.571; *p* <.001). Despite the fact that regardless of the sex, family support was relevant in order to reduce suicidal ideation (β = − 0.485; *p* <.001), as observed in Fig. 4, in general, family support had a higher impact in females (β = − 0.582; *p* <.001) than in males (β = − 0.320; *p* <.001) when it comes to reducing suicidal ideation.

**Fig. 5:**
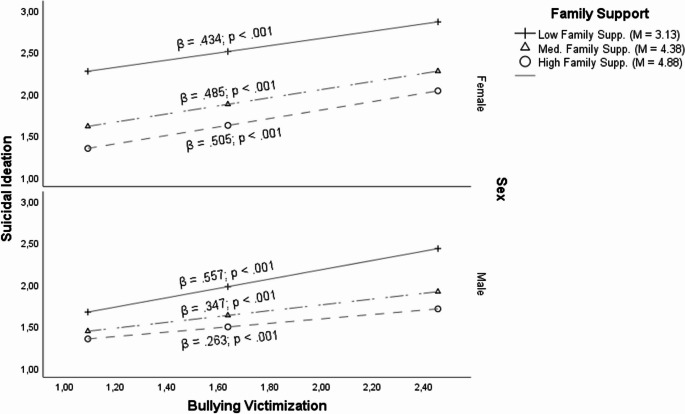
Relationship between bullying victimization and suicidal ideation when family support and sex as moderators

Relationship between Family Support and Suicidal Ideation when Sex as Moderator Nonetheless, when higher values of bullying victimization appear, this finding was reversed (β = − 0.209; *p* =.011). Therefore, it may be claimed that in spite the fact that for both sexes, family support was considered key in reducing suicidal ideation, as illustrated in Fig. 5, in bullying victimization cases males (β = − 0.168; *p* =.010) may reduce to a greater extent their suicidal ideation when there is a higher Family support in contrast to females (β = 0.041; *p* =.402).

In fact, as observed in Fig. 5, β-values of males are reduced when the family support increase, stating that higher family support values contribute to reducing suicidal ideation in higher bullying victimization cases. On the contrary, β-values of females are higher when the family support increase, stating that higher family support values contribute to enlarging suicidal ideation in higher bullying victimization cases.

Finally, with regard to the RQ3, it said:

Does teacher support moderate the relationship between bullying victimization and suicidal ideation in adolescents, and if so, to what extent individual characteristics, like age, sex or retaken a course, affect this relationship?

On this matter, as observed in Table 2, regardless of the sex, age and condition of retaking a course, higher teacher support contributes in a statistically significant way to reducing suicidal ideation (β = − 0.090; *p* <.003), in general cases, as well as in bullying victimization cases.

## Discussion

The aim of this study has been to analyze the moderating role of social support and personal characteristics (age, sex and retake a course) in the relationship between bullying victimization and suicidal ideations.

The first finding revealed that higher levels of bullying victimization had a worse impact on suicidal ideation in females than in males. This finding has been shared with other previous studies (e.g., Reed et al., 2015), but is also contrary to other studies that point out how sex did not work as moderator between bullying and suicidal ideation (e.g., Lucas-Molina et al., 2018). Hence, further research is needed in order to untangle this research question.

The second finding revealed that in general, family support was more useful for females in reducing suicidal ideation, but in bullying victimization cases, a greater family support for men was more effective in reducing suicidal ideation than in females. Despite the fact that in this model sex plays a significant role and due to it, this finding could be novel, there is some previous evidence (e.g., Baiden et a., 2019) that points out how bullying victimization is closely linked to suicide attempts when there is a lack of peer and family support, as well as when some sentiments appear (loneliness, socially hopeless, anxiety…). In fact, the vast majority of previous evidence reveal that family support works as a protective factor of the mental health (Rothon et al., 2011; Secord et al., 2018; Šmigelskas et al., 2018). Nonetheless, and although it did not occur in the present study, it should be highlighted that an excessive high family support may cause the contrary expected outcome in adolescents’ mental health (Ringdal, 2020).

In addition, the third finding revealed that the relationship between peer support and suicidal ideation was stronger for females than for males. These results shed more light to previous studies that revealed how social support played a significant buffer effect between victimization and suicidal ideation, but did not introduce to their models individual characteristics (e.g. Eze et al., 2021), except in the case of Secord’s et al. (2018) study which highlight how close peer support plays a buffer effect when dealing with negative outcomes more in girls than in boys. Our results also bear this conclusion.

Moreover, this moderation analysis revealed that the relationship between peer support and suicidal ideation was moderated by the condition of retake a course. This finding is novel as it is not a common variable analyzed in the literature, but it may be justified on the grounds that students that have retaken a course have not formed sufficiently strong social relationships with their new peers to benefit from them in order to reduce suicidal ideation.

Finally, the forth finding revealed that regardless of the analyzed personal characteristics (sex, age and retake a course), teacher support contributed to reducing suicidal ideation. This finding is novel as it adds to the model the impact of personal characteristics, but it is shared with previous findings that state how teacher support is a relevant variable for developing students’ perceived resilience (Rosenberg et al., 2018), that could be key when it comes to reducing suicidal ideation. Moreover, Conner et al. (2014) revealed that having support from a more quantity of teachers may be a stronger protective factor against academic anxiety, internalizing symptoms and physical problems linked to school stress in contrast to having just a single close relationship with a teacher.

### Theoretical and Practical Implications

All of these discoveries have significant theoretical and practical consequences. In terms of theoretical implications, these findings allow for a firmer understanding of existing theoretical models that link school victimization with suicidal ideation. More importantly, the study clearly identifies how family, peer, and teacher support operate as differential protective factors, thereby specifying which sources of support contribute most strongly to buffering suicidal ideation in victimized adolescents. This strengthens current theoretical frameworks by providing evidence of both general and source-specific protective effects. More specifically, this study makes its contribution to the list of protective factors that contribute to the reduction of suicidal ideation and attempts in the adolescent population.

With regard to practical implications, these results could have a significant utility for teaching practice. In fact, given the negative association between social support and suicidal ideation in victimization cases, policy makers and educational organizations should try to implement a syllabus for how social support should be fostered. This study contributes by specifying concrete support agents (family, peers, and teachers) and by clarifying the conditions under which each agent is most protective, which can directly guide school-based prevention programs. Furthermore, according to the World Health Organization (2020), interventions should involve building a supportive atmosphere and social networks in order to reduce youth suicide risk.

On this matter, it could be interesting to foster peer support by means of cooperative learning, as there is some evidence of how cooperative learning significantly reduces bullying prevalence and has positive effects on affective empathy (Van Ryzin & Roseth, 2019).

Secondly, it could be interesting to foster family support by means of involving families in school projects, for instance, through using different methodologies like dialogic literacy gatherings or learning interactive groups, to name but a few. In fact, there is some previous evidence of the impact of dialogic literacy gatherings with families in order to improve prosocial behaviors, like solidarity and friendship (Villardón-Gallego et al., 2018). Prosocial behavior could be an interesting construct to be enhanced in a way that they could not only improve students’ peer support, but also family welfare. The current findings specify why strengthening family support is relevant, especially for adolescents who experience bullying victimization.

Thirdly, it could be interesting to foster teachers’ support by improving the interpersonal and regulatory social context. First, teachers could improve their interpersonal social context by showing a higher concern and empathy not only for the performance of the students but also especially for the social and emotional problems that their students may present. Second, teachers could improve their regulatory social context by establishing clear and fair rules, as well as the consequences that non-compliance with the rules may entail (Villa, 1992). As a matter of fact, previous data revealed how classes with high scores of teachers’ support tend to be built less hierarchically in contrast to those with different teachers that tend to present a more hierarchical peer ecology (Hendrickx et al., 2016). Hence, teachers’ support is considered a relevant variable in order to avoid a divided class where each group plays a different role within the class context. The present study reinforces this idea by demonstrating that teacher support consistently predicts lower suicidal ideation across demographic groups, highlighting it as a universal protective factor.

Moreover, in order to reduce suicidal ideation in victims, teachers must develop and execute a very strict protocol of how to deal with bullying cases. Burger et al. (2015) expose some suggestions based on scientific data of how teachers should behave in bullying cases. Amongst the main strategies a teacher can develop, the most relevant ones are carrying out authority-based interventions (82.1% of teachers), working with the bully (44.1% of teachers), working with the victim (26.7% of teachers) and enlisting other adults like staff members at school, inform the parents, refer the matter to an administrator… (40.8% of teachers). By the same token, Potard et al. (2022) point out some coping strategies bully-victims present, such as internalizing and self-blame, rumination or cognitive distancing, to name but a few. Detecting these different coping strategies may be useful in order to develop counselling strategies for the victims of bullying. The findings of this study contribute to this practical field by indicating which forms of support are most relevant to prioritize when designing school protocols and counselling interventions aimed at reducing suicidal ideation among victimized adolescents.

### Limitations and Prospective

Finally, despite the implications given, this research has limitations that should be examined to have a broader view on the data. First, the tools used were self-report instruments; thus, the findings supplied by some individuals could have been biased due to diverse reasons (mood, significant events on the day…). As a result, future study might focus on employing different types of evaluation tools, such as instruments based on teachers or instruments based on parents. In addition, incorporating observational methods or multi-informant assessments could help reduce common-method bias and provide a more robust picture of adolescents’ social experiences.

Second, due to the cross-sectional character of the study, causality from the results cannot be established. Consequently, future research might repeat the study’s aim, assessing the various characteristics at periodic times, such as at the start and finish of an academic course. Longitudinal or prospective designs would allow researchers to examine temporal ordering, developmental trajectories, and potential bidirectional relations between victimization, social support, and suicidal ideation.

Third, data on stress problems were not obtained; consequently, these findings may vary based on the health of the teenagers. In this regard, future research should do the same analysis with other types of subsamples depending on characteristics linked with adolescent health (stress disorders, personality disorders…). Further studies could also explore whether stress-related symptoms, emotional dysregulation, or coping styles act as mediators or moderators, thereby clarifying the mechanisms through which social support influences suicidal ideation among victimized youth.

Moreover, future work may benefit from considering contextual variables—such as school climate, classroom norms, or digital forms of peer interaction—that could shape both bullying experiences and the availability of supportive relationships. Evaluating these factors could expand the ecological validity of the findings.

Despite these limitations, this paper is intended to be used as a starting point for a series of forthcoming papers analyzing the impact of the social context on the relationship between bullying and suicidal behavior. The present results thus provide a foundation for more comprehensive research programs seeking to understand how multiple layers of adolescents’ social environments jointly contribute to risk and protection.

## Data Availability

The data and material of the research can be obtained after contacting the correspondence author.
